# Use of Biomaterials in 3D Printing as a Solution to Microbial Infections in Arthroplasty and Osseous Reconstruction

**DOI:** 10.3390/biomimetics9030154

**Published:** 2024-03-01

**Authors:** Argyrios Periferakis, Aristodemos-Theodoros Periferakis, Lamprini Troumpata, Serban Dragosloveanu, Iosif-Aliodor Timofticiuc, Spyrangelos Georgatos-Garcia, Andreea-Elena Scheau, Konstantinos Periferakis, Ana Caruntu, Ioana Anca Badarau, Cristian Scheau, Constantin Caruntu

**Affiliations:** 1 Department of Physiology, The “Carol Davila” University of Medicine and Pharmacy, 050474 Bucharest, Romania; 2 Akadimia of Ancient Greek and Traditional Chinese Medicine, 16675 Athens, Greece; 3 Elkyda, Research & Education Centre of Charismatheia, 17675 Athens, Greece; 4 Department of Orthopaedics and Traumatology, The “Carol Davila” University of Medicine and Pharmacy, 050474 Bucharest, Romania; 5 Department of Orthopaedics, “Foisor” Clinical Hospital of Orthopaedics, Traumatology and Osteoarticular TB, 021382 Bucharest, Romania; 6 Tilburg Institute for Law, Technology, and Society (TILT), Tilburg University, 5037 DE Tilburg, The Netherlands; 7 Corvers Greece IKE, 15124 Athens, Greece; 8 Department of Radiology and Medical Imaging, Fundeni Clinical Institute, 022328 Bucharest, Romania; andreea.ghergus@gmail.com; 9 Pan-Hellenic Organization of Educational Programs (P.O.E.P.), 17236 Athens, Greece; 10 Department of Oral and Maxillofacial Surgery, “Carol Davila” Central Military Emergency Hospital, 010825 Bucharest, Romania; 11 Department of Oral and Maxillofacial Surgery, Faculty of Dental Medicine, Titu Maiorescu University, 031593 Bucharest, Romania; 12 Department of Radiology and Medical Imaging, “Foisor” Clinical Hospital of Orthopaedics, Traumatology and Osteoarticular TB, 021382 Bucharest, Romania; 13 Department of Dermatology, “Prof. N.C. Paulescu” National Institute of Diabetes, Nutrition and Metabolic Diseases, 011233 Bucharest, Romania

**Keywords:** biomaterials, 3D printing, antimicrobial, pathophysiology, orthopedics, bone reconstruction, implants, imaging

## Abstract

The incidence of microbial infections in orthopedic prosthetic surgeries is a perennial problem that increases morbidity and mortality, representing one of the major complications of such medical interventions. The emergence of novel technologies, especially 3D printing, represents a promising avenue of development for reducing the risk of such eventualities. There are already a host of biomaterials, suitable for 3D printing, that are being tested for antimicrobial properties when they are coated with bioactive compounds, such as antibiotics, or combined with hydrogels with antimicrobial and antioxidant properties, such as chitosan and metal nanoparticles, among others. The materials discussed in the context of this paper comprise beta-tricalcium phosphate (β-TCP), biphasic calcium phosphate (BCP), hydroxyapatite, lithium disilicate glass, polyetheretherketone (PEEK), poly(propylene fumarate) (PPF), poly(trimethylene carbonate) (PTMC), and zirconia. While the recent research results are promising, further development is required to address the increasing antibiotic resistance exhibited by several common pathogens, the potential for fungal infections, and the potential toxicity of some metal nanoparticles. Other solutions, like the incorporation of phytochemicals, should also be explored. Incorporating artificial intelligence (AI) in the development of certain orthopedic implants and the potential use of AI against bacterial infections might represent viable solutions to these problems. Finally, there are some legal considerations associated with the use of biomaterials and the widespread use of 3D printing, which must be taken into account.

## 1. Introduction

The emergence of additive manufacturing, most commonly known as 3D printing, has opened new possibilities in different scientific fields (e.g., [1,2,3,4,5,6]), including medicine (e.g., [7,8,9,10,11,12]). The 3D printing approach enables the building of a three-dimensional geometrical object layer-by-layer, guided by computer-aided design (CAD)/computer-aided manufacturing (CAM) software [13,14,15,16,17,18]. Improvements established in CAD/CAM software allow the producer to include the usual post-processing steps, such as milling, in the initial design, reducing manual post-processing stages that often increase errors in the final build [19]; the medical applications of 3D printing are ever-expanding, opening new frontiers in personalized medicine [20,21,22,23].

There are currently several 3D printing technologies available, classified under the ISO/ASTM52900-21 standard [24]. Amongst the many different applications of such techniques in medicine, a prominent one, perhaps the most prominent, is the development of prostheses for a variety of surgical procedures; such prostheses improve both the outcome and quality of life of the patients [25,26,27,28] by solving or at least mitigating some of the problems associated with surgical interventions.

Specifically in orthopedic surgeries, a notable problem involving the use of prostheses is the associated bacterial infections [29,30,31]. The use of artificial implants is already complex enough, given that they must have proper mechanical and structural properties, along with physicochemical compatibility with the natural bone tissue; different materials may be used for different applications, alone or combined [32,33,34,35]. However, regardless of all other considerations, the emergence of a microbial infection can lead to implant failure and even result in amputations and increases in mortality [36,37]. Such infections are most commonly the result of microbial biofilm formation on the surface of the implants [38,39,40]. Such bacterial biofilms oftentimes prove excessively resistant both to the host’s immune system and even to antibiotics [41,42]. Ideally, materials used in surgical prostheses must be both habitable by bone-forming cells and also have suitable anti-adhesive properties, so as to prevent biofilm formation [43]. Moreover, these materials must present an optimal rate of biodegradation, to create space for new bone formation and to exhibit osteogenic, osteoconductive, and osteoinductive properties for proper integration into the body, as a balance between the pore sizes of the build and the rate of biodegradation needs to be found [44,45,46,47,48,49].

In the context of this review, we will present the current consensus on the problem of orthopedic prostheses-associated bacterial infections and the current developments in the antimicrobial properties of biomaterials used in 3D printing to produce orthopedic materials.

## 2. Microbial Infections in Orthopedic Prostheses

The risk of microbial-related complications during orthopedic surgery is a major concern oftentimes necessitating pre-emptive systemic use of wide-spectrum antibiotics and proper debridement [50]. Despite this vast array of protective measures, the prevalence of surgical site infections remains significant, making up 12–16% of all nosocomial infections [51], and it has been suggested that absolute prevention might just not be feasible [52]. It was shown that the occurrence of surgical site infections, particularly for those patients who undergo multiple operations, and especially deep site infections, are more common in orthopedic surgery when compared with the traumatological department of the same clinic [53].

As such, surgeons go to great lengths to ensure the safety of established methods and to find new ones that pose less of a risk to patients. Indicatively, antibiotic bone cement is widely used to minimize the risk and recent findings indicate that antimicrobial tapes can be similarly effective [54]. Moreover, the implants used in oral and orthopedic surgery are made of alloys like stainless steel and titanium in order to prevent biofilm-associated infections [55,56]. This composition is essential in avoiding prosthetic infections which can necessitate long-term administration of antimicrobial regimens and even removal of the prosthetic, burdening both the patient and the healthcare system with additional hospitalizations [57]. Moreover, it was observed that different surfaces of titanium can induce anti-inflammatory responses mediated by the activation of M2-like macrophages that increase the level of interleukins 4 and 10 (different for smooth or rough titanium), creating a microenvironment with immunological properties, optimal for the healing response in patients with 3D-printed prostheses coated with titanium [58]. Although metal prostheses come with many advantages, from manufacturing to in-body responses, disadvantages can occur in the case of patients allergic to metals [59]. Research has likewise been conducted in regard to the efficacy of the incorporation of capsular traction sutures and it was concluded that they carry a low risk of colonization and thus can be used quite safely in hip arthroscopic surgery [60]. Other researchers have found out that the application of a cyanoacrylate-based skin sealant, called InteguSeal, seems to be beneficial during trauma surgery albeit without the results being conclusive [61]. At any rate, the standard operating room cleaning practices are most likely efficient in dealing with both infectious as well as non-infectious cases, as demonstrated by Balkissoon et al. [62] whose results suggest that conducting surgery on the former type of patient does not compromise a subsequent surgery on the latter that is conducted in the same room.

At the same time, other aspects of orthopedic surgery, such as the utilization of certain tourniquets [63] or sterile stockinettes [64], have been identified as possible sources of contamination. Similarly, a systematic review that examined implant contamination in spinal surgery by going over thirty-five studies deduced that even though intraoperative contamination can be reduced by taking certain safety measures, preoperative contamination through the utilization of single-use implants has not been shown to yield notable positive results [65]. Conversely, current intraoperative implant prophylaxis practices seem to not be as thorough as they could be, and thus new recommendations are being made [66].

The main risk stems from *Staphylococcus epidermidis*, *Staphylococcus aureus*, *Staphylococcus pettenkoferi*, and *Micrococcus luteus* bacteria [66] while *Proteus mirabilis* and *Citrobacter koseri* have also been implicated [60]. *Staphylococcus aureus* in particular may be responsible for septic arthritis and osteomyelitis, two severe conditions [29]. *Corynebacterium* spp. were also found to be present in a notable percentage of orthopedic patients belonging to a certain cohort, particularly *C. striatum* and *C. tuberculostearicum* [67]. The presence of *Corynebacterium* in an orthopedic setting has also been confirmed by the research of Walsh et al. [68] who traced it on tourniquets alongside coagulase-negative staphylococci, *Aerococcus viridans*, and even *Bacillus* spp. *B. anthracis* and *B. cereus*, which are pathogens that can cause lethal infections [69]. Their ability to form spores, thus protecting themselves from adverse environmental conditions and becoming impervious to the action of disinfectants, is a major factor contributing to the burden of disease [70,71]. In regard to the aforementioned *M. luteus*, infections caused by this germ are infrequent and occur mainly in immunocompromised patients, in the form of bloodstream infections [72]. Moreover, it has a notable presence on the mobile phones of medical personnel, ranking second after coagulase-negative staphylococci, with *Bacillus* spp. coming in at third place. This is an important finding as these devices can serve as a source of infection in orthopedic surgeries, potentially leading to surgical site infections [73].

Among the microorganisms mentioned, *Staphylococcus aureus* remains by far the most commonly encountered causative agent [74,75], accounting for two-thirds of all pathogens in orthopedic implant infections [29] and originating both from exogenous sources and due to the patient being a carrier of *S. aureus* when the surgery takes place, which actually constitutes a risk factor for infection [76]. It should be mentioned however that the diversity of microbes encountered is variable depending on the wound’s localization, with *E. coli* being responsible mainly for infections following visceral surgery [75]. Alarmingly, the research of Wolcott et al. [77] indicates that a plethora of other microorganisms can be involved as they identified anaerobic bacilli and most notably two previously uncharacterized Bacteroidales. A similar microorganism, *B. fragilis*, despite having a beneficial role for the host while in the gut [78,79], can cause infections when it finds its way out of the gastrointestinal tract [79], oftentimes resulting in notable bacteremia and abscess formation [78]; it is also commonly associated with polymicrobial infections [80]. Both its drug resistance [78,81] and its virulence, attributed in large part to its encapsulation, are notable; it therefore poses a significant threat [80].

The problem is exacerbated by the fact that several microbes, such as the extended-spectrum beta-lactamase Enterobacteriaceae, including the already mentioned *E. coli* and *K. pneumoniae*, account for many of these prosthetic infections and expose the patients to the risks of extensive antibiotic therapy and prosthesis removal, as discussed above, due to them being particularly resilient in the face of any attempts to eliminate them [82]. The latter is an opportunistic pathogen that is so widespread around the world that it makes up one-third of all Gram-negative bacterial infections [83]. Not only can it be the etiologic agent of severe nosocomial infections [84], but several strains have developed resistance to even last-line antibiotics [84,85].

However, there is great concern regarding *S. aureus* which is notorious for its MRSA strains that are characterized by significant morbidity and mortality and are very prevalent in the community as well as the nosocomial setting, wherein orthopedic patients find themselves; at the same time, these infections are very hard to treat [86]. Unfortunately, the same can be said about *C. striatum* which is becoming an important determinant of potentially lethal infections in the nosocomial setting, owing in large part to its biofilm formation capacity, with a number of MDR (multi-drug resistance) strains having been identified [87]. Other bacteria, like *A. viridans*, show a variable level of resistance, with several strains being impervious to the action of erythromycin, tetracycline, and minocycline, while resistance to other antibiotics like chloramphenicol and streptomycin was noted only in a single strain [88].

## 3. Biomaterials Compatible with Antibiotic Infusion

**Figure 1:**
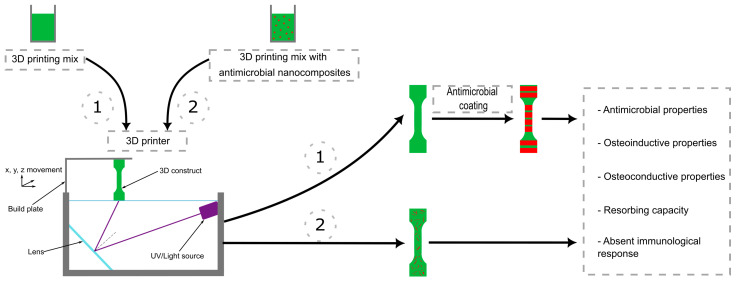
Antimicrobial substances can be either applied as a coating after printing the final construct (1) or inoculated directly into the initial mix (2). The figure also presents a summary of the properties of the models resulting from this method. The 3D printing technique given as an example in this figure is based on the principles of vat-photopolymerization (DLP, SLA).

We can classify biomaterials as organic or inorganic, based on their nature; this is purely a classification scheme however, as it does not affect their suitability for 3D printing or their range of applications. While the chemical processing of organic materials is rather more complex, requiring polymerization of the organic compound to reach the final, 3D printable, synthetic form, the selection of the optimal biomaterial for 3D printing is performed in regard to its properties and the specific requirements of the application [24]. In this review, the inorganic materials discussed are β-tricalcium phosphate (β-TCP), biphasic calcium phosphate (BCP), hydroxyapatite, lithium disilicate, and zirconia; the organic materials are polyetheretherketone (PEEK), poly(propylenefumarate) (PPF), and poly(trimethylene carbonate) (PTMC). Their current uses as biomaterials are summarized in Table 1. The printing processes and the ways in which they are combined with antimicrobial substances are represented in the figure below (Figure 1).

### 3.1. Beta-Tricalcium Phosphate (β-TCP)

Of the four different forms of tricalcium phosphate, its beta form is of interest in 3D printing applications as it is both heat-stable and printable [125]. It is currently regarded as being of prime importance in bone graft construction [126]. Bone grafts made of β-TCP using 3D printing are suitably porous and strong [47]. Its potent bioactive properties comprise osteoconduction [47] and osteoinductivity [126], gradual biodegradation [48], and reasonably low cytotoxicity [127]. A few studies showed that compared with other biocompatible, 3D-printable materials, β-TCP presents rapid degradation in vivo, which may produce undesirable mechanical features, but these could be overcome with different combinations or metal loads [128].

This compound has already been successfully combined with antibiotics, namely gatifloxacin [129], ciprofloxacin [130], tetracycline [131], vancomycin [132,133,134], and gentamycin [135]. It also displays antimicrobial properties when combined with metals, namely zinc [136], boron nitrite nanotubes [137], iron [138], and silver alone [139,140] or as a hydrogel component [141]; notable antimicrobial properties were also observed when it was combined with chitosan [142,143]. Other combinations with glass [144,145,146] or other artificial compounds [147] have also been successfully tested for their antimicrobial capacity (Table 2).

### 3.2. Biphasic Calcium Phosphate (BCP)

This is a bioceramic, comprising hydroxyapatite and β-TCP; their ratio, which can vary depending on the needs, determines the properties of the final product [98]. The particulars of the bioactive properties of this material are a reflection of those of its constituents [44,148,149]. BCP exhibits good cytocompatibility and low cytotoxicity and is currently regarded as a prime choice for bone scaffold production [45,46,150]. The morphology of the builds and their influence on their bioactive properties is also a notable aspect, along with the dimension of the pores inside the construct [44,151].

An experimental BCP formulation has been proven capable of reliably eluting antibiotics [152]; combinations with silver ions [153,154] or chitosan [143,155] have also demonstrated antimicrobial properties. In the research of Chen et al. [143], even though no testing was performed on bacterial cells, their compound promoted osteoblast differentiation and activity; this can have important implications given the interplay between osteoblasts and bacterial infections [156,157,158,159] (Table 3).

### 3.3. Hydroxyapatite (HAP)

Hydroxyapatite, with the chemical formula ${Ca}_{10}{\left(OH\right)}_{2}{\left({PO}_{4}\right)}_{6}$, is a basic component of the structure of human bones [160,161,162]. Apatite also occurs in nature [163,164,165], sometimes as inclusions in gems [166] or in association with other minerals [167,168]. As a biomaterial, it has very good properties [169], and it is hoped that in combination with metal implants, it will be able to increase the biointegration of the latter [170]. The potential of hydroxyapatite as a biomaterial is indeed immense [24] and is associated with its capacity to promote cellular integration and responsiveness [171].

When hydroxyapatite, as a biomaterial, was doped with nickel, tin, and molybdate ions [172], with zinc [173,174], cobalt [175], copper [176], titanium [177], tellurium [178], magnesium [179,180], silver nanoparticles [181,182], or a zinc and gallium combination [183], the results were promising, in that the addition of a small quantity of metals was enough to render the material active against several microorganisms. Another combination with a number of metals also proved effective [184] in this role. It has also been proven possible and successful to combine hydroxyapatite with ciprofloxacin [185] and with ciprofloxacin, dexamethasone, and metal ions [186] and chitosan [187,188]. Finally, some other combinations have been tested in this role, namely with baicalein [189], a plant flavonoid with noted antibacterial effects [190], a composite hydrogel–gelatin material with Ag nanoparticles [191], lactoferrin [192], a molecule with recently recognized promising properties [193], and alginic acid [194] (Table 4).

### 3.4. Polyetheretherketone, Poly(Propylene Fumarate), and Poly(Trimethylene Carbonate)

Polyetheretherketone (PEEK) has many favorable characteristics, which render it a suitable choice for use in orthopedic prostheses [195]. This material can be used for 3D printing [196,197,198] combined with computer-aided design (CAD) surgical planning, which has recently been gaining favor, especially in craniomaxillofacial reconstruction [199,200]. While it is stable from a chemical standpoint [201], its biological properties are associated with relatively poor osseointegration [202,203]. The addition of carbon fibers in PEEK can improve some of its properties [204,205]; nonetheless, it is still associated with some cytotoxicity [206,207].

There has been some research discussing PEEK implant infections [197,208] but strategies have already been tested on how to improve its antibacterial properties. It has been found that the surface modification of PEEK with sulfuric acid alone [209,210] or in combination with some metals [211,212] has a noted antibacterial effect in vitro; some such combinations even demonstrated this effect in vivo [209,212]. The sulfonation of composite materials containing PEEK exhibited promising antibacterial properties [213,214,215]; the coating of PEEK with antibiotic substances has also been applied successfully [216,217,218,219] (Table 5).

Poly(propylene fumarate) (PPF) has a fumaric acid base structure, opening up a number of potential medical applications [220]. Although it is neither osteoconductive nor osteoinductive, and therefore does not promote tissue regeneration, it has a number of other advantageous biological properties, such as great resorption [117,221]. When combined with various amounts of polyethylene glycol-functionalized graphene oxide (PEG-GO), it exhibits antibacterial action with no commensurate increase in cytotoxicity [222]. Nonetheless, there remain some considerations and challenges regarding its adaption as a biomaterial for 3D printers [117,223] (Table 5).

Poly(trimethylene carbonate) (PTMC), which is derived via ring-opening polymerization [120,224,225], exhibits increased compatibility with body fluids [225]; it has no intrinsic bioactivity but it can be suitably modified for medical engineering [122]. When combined with vinyl pyrrolidone (NVP), carboxymethylcellulose (CMC), and poly(lactic-co-glycolic acid) (PLGA), it also exhibits antimicrobial activity [226] (Table 5).

**Table 5:** Modifications of PEEK, PPF, and PTMC with antimicrobial properties.

Biomaterial	Modification	Dosage and Compounds	Setting	Tested Microorganism	Year	Reference
Polyetheretherketone (PEEK)	Antibiotic coating and combinations	Ag nanoparticles and gentamycin on PEEK surface	In vitro	*S. aureus*, *E. coli*	2018	[216]
		Dexamethasone and minocycline liposomes on PEEK surface	In vitro, in vivo	*S. mutans*, *P. gingivalis*	2019	[217]
		Gentamycin sulfate (5 mg/mL)	In vitro, in vivo	*S. aureus*, *E. coli*	2020	[218]
		Dopamine hydrochloride (2 mg/mL) and gentamycin sulfate (3 mg/mL)	In vitro, in vivo	*S. aureus*, *E. coli*	2021	[219]
	Composite material from sulfonation by concentrated sulfuric acid	PEEK sulfonation by concentrated sulfuric acid	In vitro	*S. aureus*, *E. coli*	2020	[213]
		PEEK combination with nanoporous tantalum pentoxide and subsequent treatment by concentrated sulfuric acid	In vitro, in vivo	*S. aureus*, *E. coli*	2021	[214]
		PEEK combination with porous Ta nanoparticles and genistein	In vitro	*S. aureus*, *E. coli*	2022	[215]
	Surface modification	PEEK sulfonation by concentrated sulfuric acid	In vitro, in vivo	*S. aureus*, *E. coli*	2016	[209]
		Creation of sulfonate PEEK biofilms	In vitro	*S. mutans*, *E. faecalis*	2017	[210]
		Surface modification with concentrated sulfuric acid and Ar	In vitro	*S. aureus*, *E. coli*	2018	[211]
		Surface modification with concentrated sulfuric acid and Cu nanoparticles	In vitro, in vivo	*S. aureus* (MRSA)	2019	[212]
Poly(propylene fumarate) (PPF)	Combinations with other materials and compounds	Polyethylene glycol-functionalized graphene oxide (PEG-GO)	In vitro	*S. aureus*, *S. epidermidis*, *P. aeruginosa*, *E. coli*	2016	[222]
Poly(trimethylene carbonate) (PTMC)		N-vinyl pyrrolidone (NVP), carboxymethylcellulose (CMC) and poly(lactic-co-glycolic acid) (PLGA)	In vitro	n/a–theorized antibacterial use	2015	[226]

### 3.5. Zirconia and Lithium Disilicate

Zircon dioxide, also known as zirconia $\left({ZrO}_{2}\right)$, occurs naturally as the mineral baddeleyite [227,228] and has excellent mechanical properties [229]; it is considered as both the most durable and aesthetically acceptable prosthesis [230,231,232]. Its biochemical and physicochemical properties justify its extensive use [231,233,234] considering its lack of bioactive properties [124]; nonetheless, there are some drawbacks associated with its 3D printing uses [231,235,236,237]. A few of the properties of zirconia, such as its low cytotoxicity and resistance to colonization of bacteria, and also good 3D printability, make this material relevant for review [234,238]. Zirconia has been tested for antibacterial action, when nanomodified [239], with a chitosan-containing surface modification [240], or when combined with Ag nanoparticles [241]; all such tests have proved successful.

Lithium disilicate is a glass-ceramic material with the chemical formula ${Li}_{2}{Si}_{2}O_{5}$ and has a biphasic crystalline structure [242]; it is currently mostly used in dental operations [106]. New 3D printing techniques have increased its usefulness and potential [243,244,245]. The combination of lithium with glass nanoparticles has exhibited some positive antibacterial results [105] (Table 6).

## 4. Discussion

### 4.1. Critical Insight on Available Data Regarding Antimicrobial 3D-Printed Implants

From all the aforementioned studies, it is implied that the biomaterials utilized must have properties that can both mimic the characteristics of the replaced/reconstructed tissues and have antimicrobial properties so as to mitigate the risk of failure of the operation. The proper selection of materials and their most beneficial combination is paramount; such an endeavor can be undertaken by using a comprehensive approach to develop biomaterials for 3D printing [24]. Implant-associated infections are ever increasing as the sheer number of such surgeries [246], the relevant burden of disease [247,248], and the need for revision surgeries [249] also increases. Therefore, the need to develop new techniques, based on current technologies, is paramount [250].

Both biomaterials and techniques and methodologies associated with their production and application are increasing (e.g., [251,252,253,254,255,256,257,258,259]). As can be seen from the information heretofore presented, there exist numerous options, particularly for developing 3D printing-adapted biomaterials with antimicrobial properties. Many experiments are focused on the combination of existing biomaterials with metal nanoparticles. Indeed, the antimicrobial potential of metal nanoparticles has been studied in detail by numerous researchers (e.g., [260,261,262,263,264,265,266,267,268,269]). Based on recent evidence, metal nanoparticles may also have an important role to play in the diagnosis and even treatment of cancer [270,271]; this can be important in cases of bone degeneration and even fracturing due to cancer [272,273]. A typical example is the case of osteosarcoma, a primary bone malignancy [274,275] where sometimes the only therapeutical avenues include allografting and autografting along with metallic prostheses [276]. Despite all these research efforts, it must be noted that there is still a lack of a complete understanding of the potentially toxic effects of some metal nanoparticles [277,278].

In contrast to inorganic materials that are more commonly used in 3D printing, recent studies have focused on organic materials, resulting from polymerization, which have notable properties, such as poly(methyl methacrylate) (PMMA), PTMC, PEEK, and PPF [24]. PPF, unlike the other materials presented in this review, does not exhibit bioactive properties, but it compensates through its superior mechanical properties and the possibility of creating a structure with unique geometries and the optimal porosity that can later be coated or loaded with antibiotics [279].

On the other hand, PMMA and PTMC (methacrylate-based polymers) present better utility in building a 3D model that mimics soft tissues [280,281]. The use of antibiotics together with these materials has not yet been researched, but adding antimicrobial substances in the final structures, for example, as a 3D-printed meniscus, could be a possibility in the future [120].

PEEK scaffolds are known to imitate the mechanical aspects of cancellous bone and also exhibit angiogenic properties, which can be enhanced with different metal-coatings such as magnesium [282]. Compared with other materials, there are no significant differences regarding the mechanical or bioactive properties but depending on the purposes of the research or the compatibility with 3D printers, a larger array of materials offers more flexibility for projects.

Quite a number of studies have focused on combinations of biomaterials with chitosan, an abundant biopolymer derived from a number of organisms [283], which has many positive properties [284,285,286,287] in addition to its more important, in the context of this paper, antimicrobial ones [288,289]. Given that the most recent research regarding the antimicrobial potential of chitosan-containing combinations and nanoparticles has demonstrated encouraging results [290,291,292,293], one can only imagine the potential of its incorporation in the prosthetics and implants field.

Another avenue, which of course has been extensively studied, is the combination of biomaterials with antibiotics. This is only natural, given that antibiotics still represent the most potent medical intervention against bacterial infections [294,295]. While the combination of biomaterials with antibiotics has been steadily gaining traction [296,297,298,299], there are still some problems with such applications. An anticipated problem is the resistance to antibiotics which is characterized by the ineffectiveness against an infection by resistant bacteria, or the creation of resistance due to the pre-emptive use of antibiotics.

We have mentioned that the most common pathogens in orthopedic implant infections are *Staphylococcus aureus*, *Escherichia coli*, and *Klebsiella pneumoniae*. For *S. aureus*, over 30% of strains are reportedly resistant to some common antibiotics [300]; indeed, there are numerous mechanisms reported to be associated with such antibiotic resistance in this bacterial species [301]. For *E. coli*, there is likewise a trend of emerging resistance based on recent studies [302,303] and the same can be said for *K. pneumoniae* [304,305]. So, a problem arises regarding the selection of antibiotics to be incorporated into the prostheses. What if there are resistant bacterial strains? Perhaps a solution would be the pre-emptive use of very powerful antibiotics such as vancomycin [306]; however, there are already bacterial strains resistant even to this drug [307,308,309], and the injudicious use of vancomycin may be by itself a cause of resistance emergence [310].

Regarding the enterococci, *E. faecium* and *E. faecalis* are the most relevant species from a clinical point of view [311] since they account for a notable part of the infections encountered in the nosocomial setting [312]. Not only can such cases be potentially life-threatening [313,314], but our means of curing them are being limited as vancomycin-resistant enterococci (VRE) strains are emerging [315]. Similarly, bacteria of the *Pseudomonas aeruginosa* species are responsible for a considerable number of nosocomial infections [316], both localized and systemic, which not only can be life-threatening [317], but may also be difficult to handle as resistant *P. aeruginosa* strains are becoming more prevalent [318,319]. *Streptococcus anginosus*, the official name of a group of bacteria commonly referred to as *S. milleri*, shows notable variety regarding its hemolytic, physiological, and serological characteristics, making its identification challenging in the laboratory setting [320]. It is clinically relevant as it can cause severe infections, particularly purulent ones [320]. Porphyromonas is most commonly associated with periodontitis, but it can also cause severe systemic infections [321,322] and has even been implicated in cognitive impairment [323] and carcinogenesis [321,324,325].

*Streptococcus mutans* is mostly known as an important cause of dental plaque formation, with its ability to form biofilms playing a critical role in its pathogenicity [326,327]. However, it can cause other serious conditions, such as life-threatening endocarditis [326] and carcinogenesis [327]. Salmonella is a major etiological agent of foodborne pathologies that is a cause for concern for global public health [328,329]. It has a characteristic diversity when it comes to serovariability, having over 2600 serotypes [330], as well as antigenic variability [331]. Its virulence and mortality rates are not to be underestimated [332], as many strains exhibit antibiotic resistance [333,334]. *S. enteritidis* is among the most frequently encountered species, and is mainly found in chicken eggs [335]. *Shigella dysenteriae* is a common causative agent of diarrhea, hemorrhagic colitis, and hemolytic uremic syndrome [336], with a wide arsenal of virulence factors at its disposal [336,337]. Antibiotic resistance is a concern in this case as well [338]. Finally, the rarely mentioned *Actinobacillus actinomycetemcomitans* is normally a part of the physiological flora of the oral cavity; it is, however, capable of causing periodontitis as well as systemic pathologies [339,340,341], such as coronary artery disease in the case of serotypes b and c [340]. It is also notable for its ability to evade the immune system [342] and its very potent leukotoxin [339].

Still on the subject of infections, we must note that while bacteria account for the majority of orthopedic infections, there are other pathogens of concern. For example, a case report by Soukup et al. [343] mentions the appearance of toxocariasis as a post-surgical complication after transthoracic spine surgery. But, apart from such rare incidents, other applications may prove useful; a prominent case might be the surgical removal of cysts of *Echinococcus granulosus* from the spine [344]. However, the surgical removal of the cysts may sometimes present complications [345]; the removal might only be partial [346] or the resultant spillage may lead to secondary echinococcosis [347,348]. In such cases, prostheses associated with proper drugs, namely albendazole, mebendazole, and perhaps praziquantel [349], may be useful. Another incidence of parasitic infection may occur in patients who are immunosuppressed in the course of rheumatoid arthritis treatment—such a case has been reported by Trigkidis et al. [350]. Perhaps, in such particular cases, and given that rheumatoid arthritis frequently necessitates the use of orthopedic prostheses [351], the incorporation of antimonial drugs, which have a proven anti-leishmanial effect [352], may prove useful.

A minor consideration, compared to bacterial infections, is fungal infections in orthopedic implants. Still, research has been conducted regarding the role that microorganisms like *Candida* spp. and *Aspergillus* spp. play in hip prosthetic joint infections, a condition most commonly associated with *Staphylococcus* spp. [353,354]. Fungi can indeed be the causative agent of such an infection, mostly owing to *Candida* spp., and, occasionally, they even coinfect the patient alongside bacterial pathogens [353,354]. Although this complication arises infrequently, it causes a severe condition that requires multidisciplinary action to be properly dealt with [353,354]. The combination of biomaterials with common antifungal agents, such as triazoles and amphotericin formulations [355,356], should be studied in the future.

In general, the development of biomaterials suitable for bioprinting can reduce, or even hopefully eliminate, the need for bone allografts and the management of the associated immune response [357,358]. Perhaps the most potent material for this purpose is hydroxyapatite [359], with BCP and β-TCP being less resilient to mechanical stress, despite having good properties overall [360]. As there are still other biomaterials better suitable for soft tissue replacement, and still other biomaterials with untapped potential [361,362], it is very possible that, in the near future, novel approaches for producing biomaterials with a potent antimicrobial action will arise.

### 4.2. Current Challenges in 3D Printing with Mixtures Containing Antimicrobial Substances

Regarding 3D printing models with antimicrobial activity, we can divide the final construct into two main groups: models with incorporated antimicrobial particles, and models with antimicrobial substance coatings or loading. In the second group, the first step is printing the 3D model separately, and in the post-processing steps, the active substances are added as a coating or loaded into the micro-pores of the construct. So, the only challenges that can occur in these cases are strictly structural aspects and later biocompatibility and bioactivity within the body, as the functionality of the build or the activity of the antimicrobial substances should not affect each other.

Many of the 3D printing technologies require high-temperature treatment of the material at the time of the printing [363]; in that case, the initial mix that contains the antimicrobial particles has to withstand these temperatures, and to still be able to exhibit antimicrobial activity after printing. Most antibiotics are thermolabile, and after thermic treatment of the mix, the bioactivity of the substances can decrease dramatically [364]. In that case, we can conclude that fused deposition modeling (FDM), a popular 3D printing technique, that uses temperatures over 80 °C is not the most efficient method in building parts with antimicrobial activity. Even if the thermic problem is managed, it is already known that the mechanical properties of the construct with antibiotics are significantly decreased compared with the constructs with no antimicrobial substances added [365].

However, metals and nanoparticles with antimicrobial activity, such as zinc, iron, copper, magnesium, and their oxides are known to maintain their antimicrobial activity after printing with the standard temperatures of different 3D printing technologies such as FDM [366]. Even though temperature is not a problem when printing with incorporated metals or metal oxides, dispersing them homogenously can still be a challenge, which can alter the final product, inducing filling defects or agglomeration of particles and causing structural instability [366].

Another method, called inkjet printing, requires high-temperature treatment only in the post-processing steps, which can be skipped if needed; in this way, the functionality of the antibiotics can be maintained [367]. With inkjet printing, it was already demonstrated that adding antibiotics does not alter the mechanical properties of the final build, but there are not many biocompatible materials that can be used with this technology; shortly, new materials may be available [368]. Antibiotics or nanoparticles with antimicrobial activity that are UV sensible cannot be printed by vat photopolymerization; however, positively charged quaternary ammonium compounds and silver–halloysite in combination with methacrylate-based polymers such as PTMC and PMMA reported good results in dentistry applications [367].

From our team’s experience with 3D printing, especially with stereolithography (SLA) technology, finding the optimal mixture of the biocompatible material and the antibiotic or antimicrobial particles is the most important goal. Different technologies require different solubilities, viscosities, and temperatures, and also, different post-processing steps. Finding ways to solubilize the antimicrobial substance in a way that the optimal viscosity is maintained and the mechanical properties of the final constructs (that usually contain pores of different sizes) are not altered are the main challenges that should be further studied.

### 4.3. Legal Considerations for 3D-Printed Antibiotic-Integrated Medical Implants

Even the most promising applications inevitably bring associated challenges. In this particular situation, aside from the previously discussed considerations, there is a medico-legal dimension to take into account. In today’s medical practice, legal proceedings regarding medical responsibility and liability have become an integral aspect of the profession. This intricacy is exacerbated by factors that go beyond the conventional patient care environment, notably the incorporation of advanced technologies [369], like 3D printing. Therefore, present-day healthcare professionals need to familiarize themselves with the applicable regulatory principles and guidelines, especially when engaging with such technologies in pursuit of innovation. In the European Union (EU), medical liability is predominantly regulated by national laws; however, specific EU directives and regulations outline the overarching principles.

The EU Clinical Trials Regulation [370] delineates the regulations for clinical studies and trials, defined in Article 2(2) as inclusive of “therapeutic strategies that deviate from the normal clinical practice of the Member State concerned”. Ensuring transparency through precise and comprehensive reporting, proportional balancing of risks and benefits, obtaining informed consent, and adhering to safety standards are pivotal for safeguarding research participants. The Medical Devices Regulation [371] focuses on the concept of medical devices, defined in Article 2(1), and relevant terms such as an “accessory of medical device” [Art. 2(2)], “implantable device” [Art. 2(5)], etc. It establishes safety and performance requirements through mandatory measures, including risk classification, conformity assessments by manufacturers, clinical evaluations, and heightened scrutiny. Furthermore, the EU Patient Rights Directive [372] and the European Charter of Patients’ Rights [373] establish a framework to protect patients’ rights in the European Union, encompassing the right to information and informed consent, access to medical records, privacy, and the right to redress in case of harm. This latter right is particularly significant, empowering patients to voice complaints and seek redress in instances of medical malpractice or dissatisfaction with healthcare services.

As for the applicability of the legal or quasi-legal documents above and their provisions in the context of the present article and to provide an extra layer of information on the connection between what is written in this article and the legal status quo, the following apply:

(a) Under no circumstance, based on the current data, can the use of antimicrobial material in 3D printing in the field of Arthroplasty and Osseous Reconstruction be considered as “normal clinical practice”, meaning the day-to-day typical medical approach. Therefore, the definition of the clinical trial as mentioned in the EU Clinical Trials Regulation seems to encompass the aforementioned notion, providing the necessary framework for the implementation of the Regulation.

(b) Additionally, the Medical Devices Regulation focusing on medical devices includes in its provisions the concept of “implanted devices”, a classification which largely, if not exactly, reflects the essence of this article, meaning both the materials used and the 3D-printed orthopedic implants under discussion.

(c) As for the EU Patient Rights Directive and the European Charter of Patients’ Rights, once patients are involved in the whole procedure, they are applicable by default, and no further clarifications are required since the patients’ rights and their protection hold great significance in the EU legal framework.

### 4.4. Future Directions and Emerging Trends

The approach of using antibiotic-laden biomaterials in a protective manner is a trending idea among research groups worldwide. However, such use has been known to be either ineffective at times, or even to promote biofilm formation and resistant infection occurrence [374]. Based on the increased capabilities and potential of medical prosthetics [375], prostheses could be outfitted with methods of releasing suitable antibiotics after the source of infection has been precisely identified and the relevant resistance profile has been determined. In addition, there exists nowadays the possibility of incorporating artificial intelligence (AI) in the development of orthopedic implants [375] and given that AI is also under research for use in combatting antibiotic resistance [376,377,378], the integration of relevant AI schemes into orthopedic implants in the future might be a viable solution.

Another avenue that can be explored to enhance the antimicrobial properties of the discussed biomaterials is phytochemicals [379,380]. Recent research has highlighted the antimicrobial potential of numerous phytochemicals (e.g., [381,382,383,384,385,386]); many purified plant compounds have been found to have antimicrobial properties, such as capsaicin [387] and other capsaicinoids [388,389], curcumin [390,391,392], kaempferol and its derivatives [393,394], catechins [395,396], turmeric [397], fucoidan [398], and other plant compounds (e.g., [399,400]). Phytochemicals have already been used as coatings for a variety of materials and for a variety of purposes. Importantly, phytochemicals can be incorporated into artificial materials to lower their potential toxicity [401]. Furthermore, the research on combinations of phytochemicals and nanoparticles has yielded promising results (e.g., [402,403,404]); nanoparticles are already being applied as drug delivery systems [405], and they have also been already combined successfully with prostheses as outlined in this paper.

Finally, a further potential avenue that warrants further exploration is the combination of the materials discussed herein with antibiotic pearls for antibiotic applications. This has been shown to ameliorate the prognosis in prosthetic surgeries by eliminating biofilms and enabling extended antibiotic action both qualitatively and temporally through the use of calcium sulphate antibiotic-added beads [406,407]. The usefulness of these beads as adjuvants had previously been mentioned and can also be corroborated by the findings of Agarwal and Healy [408]. Joint infections after arthroplasty of the knee are of particular interest as it is in this context that the debridement, antibiotic bead, and retention of the implant (DABRI) method was compared to the debridement, antibiotics, and implant retention (DAIR) method and was found to be similarly effective [409]. We would also like to note, that, in the context of antibiotic pearls in particular, and of the potential combinations mentioned in this paper in general, the potential of adverse effects, especially associated with drug pairing, is a noteworthy constraint; given that such interactions could affect absorption or toxicity [410,411,412,413], caution toward administration is deemed necessary [414].

## 5. Conclusions

The antimicrobial properties of materials adapted for 3D printing are a promising research field, and there are still many compounds and combinations that can be tested. The current tests mostly revolve around combinations of existing biomaterials with antibiotics, metal nanoparticles, and chitosan. Future research must be centered around addressing the relevant problem of antibiotic resistance and the possibility, however small, of fungal or parasitic infection.

The combination of biomaterials with phytochemicals of known antibacterial potential also represents a promising avenue of research. Last, but not least, there is a need for accurate and in-depth information on medical liability frameworks in conjunction with all the relevant EU legal documents; it is also vital to refer to the individual national rules of the EU member states.

## Figures and Tables

**Table 1:** Current principal functions of biomaterials for 3D printing discussed in this paper.

Biomaterial	Current Uses	References
Beta-tricalcium phosphate (β-TCP)	Bone defect filling and repairing, bone tissue engineering and bone scaffold manufacturing, bone grafts	[89,90,91,92,93,94,95,96]
Biphasic calcium phosphate (BCP)	Bone scaffold manufacturing, bone grafts manufacturing, tissue engineering	[47,48,97,98,99]
Hydroxyapatite	Bone tissue engineering and bone scaffold manufacturing, joint replacement surgeries	[100,101,102,103,104]
Lithium disilicate glass	Bone scaffold manufacturing, dental applications	[105,106]
Polyetheretherketone (PEEK)	Spinal cages, skull/maxillofacial defect and dental implants, joint replacements, fracture healing support plates, spinal fusions	[107,108,109,110,111,112,113,114,115]
Poly(propylene fumarate) (PPF)	Bone tissue engineering, biocompatible scaffolds	[116,117,118,119]
Poly(trimethylene carbonate) (PTMC)	Bone tissue engineering, bone tissue implants	[120,121,122]
Zirconia	Hip head prostheses, orthopedic implants, dental implants	[123,124]

**Table 2:** Modifications of β-TCP with antimicrobial properties.

Modification	Dosage and Compounds	Setting	Tested Microorganism	Year	Reference
Antibiotic coating and combinations	Gentamycin	In vitro, in vivo	n/a	1996	[135]
	260 ± 48 μg of gatifloxacine hydrate per ceramic disk	In vitro, in vivo	*S. milleri*, *B. fragilis*	2008	[129]
	1 wt.% vancomycin hydrochloride	In vitro	*S. aureus* (MRSA)	2013	[132]
	5 mg/mL concentration of vancomycin solution	In vitro, in vivo	*S. aureus*	2018	[134]
	1–5 wt.% ciprofloxacin	In vitro	*S. aureus*	2021	[130]
	300 mg vancomycin hydrochloride per 1 mL water	In vitro, in vivo	*S. aureus*	2022	[133]
	1 wt.% tetracycline	In vitro	*P. gingivalis*	2024	[131]
Metal coatings and combinations	0.49 and 1.09 wt.% Fe	In vitro	*E. coli*, *S. enteritidis*, *P. aeruginosa*, *S. aureus*	2019	[138]
	1 wt.% B nitrate microtubules	In vitro	*S. aureus*	2020	[137]
	Ag nanoparticles as part of β-TCP hydrogel	In vitro	*S. aureus*, *B. subtilis*, *P. aeruginosa*, *E. coli*	2020	[141]
	5 and 10 wt.% nanosized Ag	In vitro, in vivo	*S. aureus*, *E. coli*	2020	[139]
	1.4 wt.% Zn	In vitro	*E. faecium*, *E. coli*, *P. aeruginosa*	2021	[136]
	0.1, 1, 10 wt.% Ag	In vitro	*S. aureus* (MRSA)	2022	[140]
Combination with chitosan	2 wt.% chitosan solution (3.0 g TCP based on 10.0 g chitosan)	In vitro	n/a–theorized antibacterial use	2012	[142]
	3 g of chitosan per membrane	In vitro	n/a–theorized antibacterial use	2019	[143]
Combinations with glass or other materials	2.5 wt.% β-TCP added into a PP (core layer) solution	In vitro	*S. aureus*, *S. mutans*	2018	[147]
	Ceramic suspensions with solids content of 30% wt.%	In vitro	*S. aureus*, *E. coli*, *C. albicans*	2021	[144]
	Transparent bioglass sol used to impregnate the β-TCP scaffolds	In vitro, in vivo	*C. albicans*, *P. aeruginosa*, *S. aureus*	2023	[145]
	Bioactive glass S53P4	In vitro	*S. aureus*	2023	[146]

n/a—not available.

**Table 3:** Modifications of BCP with antimicrobial properties.

Modification	Dosage and Compounds	Setting	Tested Microorganism	Year	Reference
Antibiotic coating and combinations	Vancomycin in 90 mg loaded microparticles	In vitro	n/a	2001	[152]
Metal coatings and combinations	1.06 wt.% Ag	In vitro	*S. aureus*	2021	[154]
	Variable concentration of Ag ions	In vitro	*S. aureus*, *S. epidemidis*, *E. coli*	2023	[153]
Combination with chitosan	3 g of chitosan in each membrane	In vitro, in vivo	n/a	2019	[143]
	4 *w*/*v*% chitosan	In vitro	n/a	2022	[155]

n/a—not available.

**Table 4:** Modifications of hydroxyapatite with antimicrobial properties.

Modification	Dosage and Compounds	Setting	Tested Microorganism	Year	References
Antibiotic coating and combinations	Ciprofloxacin 30 wt.%	In vitro	*S. aureus*, *E. coli*	2019	[185]
	Ciprofloxacin	In vivo, in vitro	Gram negative and Gram-negative bacteria	2023	[186]
Metal coatings and combinations	Co replacement at 5% and 12%	In vitro	*S. aureus*, *E. coli*	2016	[175]
	0.04, 0.08, 0.16, 0.24 wt.% Te content	In vitro	*B. subtilis*, *S. aureus*, *Micrococcus* sp., *P. aeruginosa*, *Klebsiella* sp., *S. dysenteriae*, *Candida albicans*	2017	[178]
	Cu addition to specific molar ratio	In vitro	*S. aureus*, *E. coli*	2017	[176]
	Mg addition to specific molar ratio	In vitro	*S. aureus*, *E. faecalis*, *E. coli*, *P. aeruginosa*, *Candida albicans*	2019	[179]
	Ag nanoparticles in different concentrations	In vitro	*S. aureus*	2021	[181]
	Zn doping at 0.25, 0.5 and 1.0 mmol/L	In vitro	*S. aureus*, *E. coli*	2021	[173]
	Ag ions in various concentrations	In vitro	*S. aureus*, *E. coli*	2021	[182]
	Doping with Ga and Zn	In vitro	*S. aureus*, *E. coli*	2022	[183]
	Various metals	In vitro, in vivo	Various microbes	2022	[184]
	ZnO 5 wt.%	In vitro	*S. aureus*, *E. coli*	2022	[174]
	Ni, Sn, and Mo ions in 500, 1000 and 2000 ppm	In vitro	*S. aureus*, *P. aeruginosa*	2023	[172]
	Ti doping	In vitro, in vivo	Various microbes	2023	[177]
Combination with chitosan	Cellulose–chitosan–hydroxyapatite composite material	In vitro	*S. aureus* (MRSA), VRE, *E. coli*, *P. aeruginosa*	2013	[187]
	Chitosan and HAP gel at 4:6 mass ratio	In vitro, in vivo	*S. aureus*, *S. epidermidis*, *P. aeruginosa*, *C. albicans*	2016	[188]
Combinations with other materials and compounds	Ag nanoparticles at 5%	In vitro	n/a–theorized antibacterial use	2012	[191]
	10 mL lactoferrin per 50 mg of hydroxyapatite	In vitro	n/a–theorized antibacterial use	2017	[192]
	63 mg/g of baicalein	In vitro	*S. epidermidis*	2021	[189]
	Different combinations of HAP and algae	In vitro	Gram-negative, gram-positive bacteria	2021	[194]

**Table 6:** Modifications of zirconia and lithium disilicate with antimicrobial properties.

Modification	Dosage and Compounds	Setting	Tested Microorganism	Year	Reference
Lithium combination with glass nanoparticles	Different concentrations of Li_2_O were used to replace Na_2_O in the glass structure	In vitro	n/a–theorized antibacterial use	2016	[105]
Zirconia with antibacterial nanomodification	An aqueous solution of a mixture of 3Y-ZrO_2_ nanopowder and ammonium citrate (dispersant)	In vitro, in vivo	*E. coli*, *S. aureus*	2019	[239]
Zirconia with Ag nanoparticles	3 g/L silver nanoparticles	In vitro	*E. coli*, *S. aureus*	2021	[241]
Zirconia surface modification with a chitosan-containing compound	5 distinct groups, each with a different mixture	In vitro	*P. gingivalis*, *A. actinomycetemcomitans*	2023	[240]

## Data Availability

Not applicable.
